# Real-time AI prediction for major adverse cardiac events in emergency department patients with chest pain

**DOI:** 10.1186/s13049-020-00786-x

**Published:** 2020-09-11

**Authors:** Pei-I Zhang, Chien-Chin Hsu, Yuan Kao, Chia-Jung Chen, Ya-Wei Kuo, Shu-Lien Hsu, Tzu-Lan Liu, Hung-Jung Lin, Jhi-Joung Wang, Chung-Feng Liu, Chien-Cheng Huang

**Affiliations:** 1 Department of Emergency Medicine, Chi Mei Medical Center, Liouying, Tainan, Taiwan; 2 Department of Emergency Medicine, Chi Mei Medical Center, 901 Zhonghua Road, Yongkang District, Tainan City, 710 Taiwan; 3 Department of Biotechnology, Southern Taiwan University of Science and Technology, Tainan, Taiwan; 4 Graduate Institute of Medical Sciences, College of Health Sciences, Chang Jung Christian University, Tainan, Taiwan; 5 Information Systems, Chi Mei Medical Center, Tainan, Taiwan; 6 Center for Quality Management, Chi Mei Medical Center, Tainan, Taiwan; 7 Department of Nursing, Chi Mei Medical Center, Tainan, Taiwan; 8 Department of Emergency Medicine, Taipei Medical University, Taipei, Taiwan; 9 Department of Medical Research, Chi Mei Medical Center, 901 Zhonghua Road, Yongkang District, Tainan City, 710 Taiwan; 10 Allied AI Biomed Center, Southern Taiwan University of Science and Technology, Tainan, Taiwan; 11 Department of Senior Services, Southern Taiwan University of Science and Technology, Tainan, Taiwan; 12 Department of Environmental and Occupational Health, College of Medicine, National Cheng Kung University, Tainan, Taiwan

**Keywords:** Artificial intelligence, Chest pain, Emergency department, Machine learning, Major adverse cardiac events

## Abstract

**Background:**

A big-data-driven and artificial intelligence (AI) with machine learning (ML) approach has never been integrated with the hospital information system (HIS) for predicting major adverse cardiac events (MACE) in patients with chest pain in the emergency department (ED). Therefore, we conducted the present study to clarify it.

**Methods:**

In total, 85,254 ED patients with chest pain in three hospitals between 2009 and 2018 were identified. We randomized the patients into a 70%/30% split for ML model training and testing. We used 14 clinical variables from their electronic health records to construct a random forest model with the synthetic minority oversampling technique preprocessing algorithm to predict acute myocardial infarction (AMI) < 1 month and all-cause mortality < 1 month. Comparisons of the predictive accuracies among random forest, logistic regression, support-vector clustering (SVC), and K-nearest neighbor (KNN) models were also performed.

**Results:**

Predicting MACE using the random forest model produced areas under the curves (AUC) of 0.915 for AMI < 1 month and 0.999 for all-cause mortality < 1 month. The random forest model had better predictive accuracy than logistic regression, SVC, and KNN. We further integrated the AI prediction model with the HIS to assist physicians with decision-making in real time. Validation of the AI prediction model by new patients showed AUCs of 0.907 for AMI < 1 month and 0.888 for all-cause mortality < 1 month.

**Conclusions:**

An AI real-time prediction model is a promising method for assisting physicians in predicting MACE in ED patients with chest pain. Further studies to evaluate the impact on clinical practice are warranted.

## Background

Chest pain is one of the most common complaints that patients present with in the emergency department (ED) and accounts for 5 to 20% of all ED visits [1]. The causes of chest pain range from myalgia to potentially life-threatening diseases, such as acute coronary syndrome (ACS), aortic dissection, or pulmonary embolism [2]. Therefore, ED physicians often face a challenge when they need to decide on a diagnosis and disposition. Meanwhile, ED physicians may arrange a series of cardiac evaluations or an imaging study to exclude the list of differential diagnoses or even liberally hospitalize patients. However, < 10% of ED patients with chest pain are eventually diagnosed with ACS, and the liberal use of serial studies and hospitalization results in a high medical cost [3]. Therefore, developing a useful clinical prediction rule to help ED physicians with decision-making becomes an important issue.

The HEART (History, Electrocardiography, Age, Risk Factors, Troponin) score was created in 2008 to facilitate accurate diagnostic and therapeutic choices in ED patients with chest pain [4]. The original risk stratifications and suggested dispositions according to HEART score are as follows: (1) 0–3 points (2.5% for major adverse cardiac events [MACE]): an immediate discharge; 4–6 points (20.3% for MACE): implies hospitalization for clinical observation; and (3) ≥7 points: (72.7% for MACE): early invasive strategies [4]. The HEART score has the advantages of high accuracy and has been well-validated in many studies [5–7]. The disadvantage of the HEART score is that it takes time to calculate for prediction; even a calculator is available online [8], which limits its use in the over-crowded ED. There are other scores (e.g., EDACS, T-MACS, and Vancouver), which do not take substantial time to calculate [9–11]. They also force the acquisition of important data that may not otherwise be acquired. In recent years, artificial intelligence (AI), including machine learning (ML) techniques, has been used more and more often for developing prediction models and risk stratification in the ED [12, 13]. Many studies have shown that ML outperforms traditional metrics [12, 13]. In addition, ML has the advantage of handling more variables that are already available through electronic medical records (EMRs) and providing real-time feedback in terms of risk stratification to physicians if implemented in the hospital information system (HIS) [12, 13]. However, the issue about utility of ML integration with the HIS for predicting MACE in ED patients with chest pain is still unclear. We did not find any studies on this issue by searching for “acute myocardial infarction,” “chest pain,” “emergency department,” “death,” “machine learning,” “major adverse cardiac events,” and “mortality” in PubMed and Google Scholar. Therefore, we conducted this study intending to clarify this issue.

## Methods

### Study design, setting, and participants

**Fig. 1:**
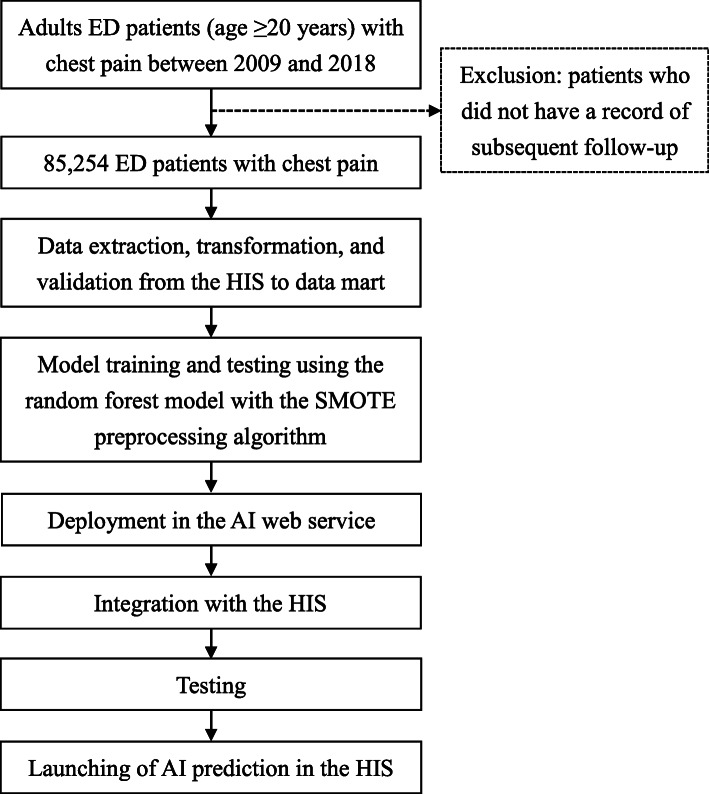
Flow chart for integrating the AI prediction model for ED patients with chest pain with the HIS. AI, artificial intelligence; ED, emergency department; HIS, hospital information system; SMOTE, synthetic minority oversampling technique

We established a multi-disciplinary team including emergency physicians, data scientists, information engineers, nurse practitioners, and quality managers for this study and AI implementation. Adult patients (age ≥ 20 years) with chest pain who visited the EDs of three hospitals (Chi Mei Medical Center, Chi Mei Liouying Hospital, and Chi Mei Chiali Hospital) between 2009 and 2018 were identified into the present study (Fig. 1). The criteria for chest pain are defined as the initial diagnostic impression according to the International Classification of Diseases, Ninth Revision, Clinical Modification (ICD-9-CM) of 786.5 or ICD-10 of R079 in the index ED visit. Patients who did not have a record of subsequent follow-up were excluded.

### Definitions of feature variables

The 14 feature variables recruited for the analyzes were the suggested predictors of MACE in previous studies as follows [5, 6, 14]: age, sex, smoking, body mass index (BMI), and past histories of hypertension (ICD-9-CM: 401–405 or ICD-10: I10-I16), hyperlipidemia (ICD-9-CM: 272.0–272.5, 277.7 or ICD-10: E78.0-E78.5, E88.81), diabetes (ICD-9-CM: 250 or ICD-10: E08-E13), chronic kidney disease (ICD-9-CM: 585 or ICD-10: N18), coronary artery disease (ICD-9-CM: 410–414 or ICD-10: I20-I25), cerebrovascular diseases (ICD-9-CM: 430–438 or ICD-10: I60-I69, G45), peripheral artery occlusive disease (ICD-9-CM: 443.9 or ICD-10: I73.9), and last laboratory data including high sensitive troponin-I, hemoglobin, and serum creatinine. The last high sensitive troponin-I is automatically captured into the AI prediction model when the ED physician pushes the AI button in the HIS when they need assistance. The past histories were defined as the diagnoses before the index visit. Electrocardiography was not recruited into this study due to the technical limitation at current stage.

### Outcome measurements

We defined two outcome measurements as follows: (1) acute myocardial infarction (AMI) (ICD-9-CM: 410–414 or ICD-10: I20-I25) within 1 month; and (2) all-cause mortality within 1 month after the index ED visit.

### Ethical statement

This study was approved by the institutional review board of the Chi Mei Medical Center. Informed consent from the patients was waived because this study is retrospective, and it contains de-identified information, which does not affect the rights and welfare of the patients.

### Data processing, comparison, and application in the HIS

First, the data were extracted from EMRs in the HIS and transformed and validated into a data mart for further analyzes. Missing and ambiguous data were defined carefully by the group meeting including emergency physicians, data scientists, information engineers, nurse practitioners, and quality managers: (1) delete the data if the feature variable could not be estimated (e.g., missing “sex”) or many feature variables are missing; and (2) add average value if the missing feature variable could be estimated (e.g., missing “BMI”). Second, we used a random forest model with the synthetic minority oversampling technique (SMOTE) preprocessing algorithm to predict AMI < 1 month and all-cause mortality < 1 month. Third, we compared the accuracy, precision, sensitivity, specificity, F1, and area under the curve (AUC) among random forest, logistic regression, support-vector clustering (SVC), and K-nearest neighbor (KNN) models. We divided the population into training and testing cohorts representing 70 and 30% of the patients, respectively. Fourth, we implemented the predictive model, by using random forest with SMOTE, in the HIS. After one month of testing and validation, we launched the AI prediction model in the HIS to assist ED physicians with decision-making in real time. Using the same data collection tool, we also prospectively identified new ED patients with chest pain between Jan 1, 2019 and Oct 31, 2019 after implementation of the AI prediction model to validate its accuracy.

## Results

In total, 85,254 ED patients with chest pain in three hospitals between 2009 and 2018 were identified into the present study. The mean ± standard deviation (SD) age was 57.7 ± 17.9 years, and the proportion of males was 55.3% (Table 1). The proportion of the three age subgroups were age 20–34 (12.8%), age 35–49 (19.5%), age 50–64 (30.0%), and age ≥ 65 (37.7%). The proportion of smokers was 21.8% and mean ± SD of BMI was 24.8 ± 3.7. The medical histories of ED patients included hypertension (47.6%), hyperlipidemia (21.1%), diabetes (26.2%), chronic kidney disease (8.2%), coronary artery disease (38.3%), cerebrovascular disease (15.1%), and peripheral arterial occlusion disease (3.6%). The mean ± SD of high sensitive troponin-I, hemoglobin, and serum creatinine were 113.9 ± 2603.2 pg/mL, 13.1 ± 2.1 mg/dL, and 1.4 ± 2.4 mg/dL, respectively. AMI < 1 month was 20.3%, and all-cause mortality < 1 month was 0.3%.

**Table 1:** Demographic characteristics, medical histories, and adverse outcomes within one month in ED patients with chest pain

Variable	Total patients (*n* = 85,254)
Age (years)	57.7 ± 17.9
Age subgroup (%)	
20–34	12.8
35–49	19.5
50–64	30.0
≥ 65	37.7
Sex, %	
Female	44.7
Male	55.3
Smoking	21.8
BMI	24.8 ± 3.7
Medical histories (%)	
Hypertension	47.6
Hyperlipidemia	21.1
Diabetes	26.2
Chronic kidney disease	8.2
Coronary artery disease	38.3
Cerebrovascular disease	15.1
Peripheral arterial occlusion disease	3.6
Laboratory data	
High sensitive troponin-I (pg/mL)	113.9 ± 2603.2
Hemoglobin (mg/dL)	13.1 ± 2.1
Serum creatinine (mg/dL)	1.4 ± 2.4
Outcome within one month (%)	
AMI	20.3
All-cause mortality	0.3

Data are presented as mean ± SD or percent. *ED* emergency department; *SD* standard deviation; *BMI* body mass index; *AMI* acute myocardial infarction

The random forest model with the SMOTE preprocessing algorithm showed that the AUCs for predicting AMI < 1 month and all-cause mortality < 1 month were 0.915 and 0.999 (Table 2). Tree-based estimators were used to compute the feature importance value, which helps to discard irrelevant features [15]. In our random forest model, we included all 14 feature variables based on the feature importance values (Supplement Figure 1 and Supplement Figure 2) and expert judgment. Comparisons of predictive accuracies among the random forest, logistic regression, SVC, and KNN revealed that the random forest model had the best AUC, accuracy, precision, sensitivity, specificity, and F1 of all the models (Table 3). In the prediction of AMI < 1 month, SVC has the poorest AUC of 0.631. Logistic regression has the poorest AUC of 0.716 in the prediction of all-cause mortality < 1 month. We integrated the AI prediction model with the HIS to assist the ED physicians in real-time decision-making. The time taken to generate prediction results is < 1 s when the ED physician pushes the AI button (Supplementary Figure 3).

**Table 2:** Evaluation report using the random forest model with the SMOTE preprocessing algorithm on the adverse outcomes in ED patients with chest pain

Outcome	Number	Negative outcome	Positive outcome	Number after imbalanced processing (over sampling)	Accuracy	Precision	Sensitivity	Specificity	F1	AUC
AMI < 1 month	85,254	67,921	17,333	135,842	0.915	0.916	0.915	0.882	0.915	0.915
All-cause mortality < 1 month	85,254	85,040	214	170,080	0.999	0.999	0.999	0.999	0.999	0.999

*SMOTE* synthetic minority oversampling technique; *ED* emergency department; F1, 2 x (precision x recall/precision + recall); *AUC* area under the curve; *AMI* acute myocardial infarction

**Table 3:** Comparisons of predictive accuracies among random forest, logistic regression, SVC, and KNN models for adverse outcomes of ED patients with chest pain

Outcomes and predictive models	Accuracy	Precision	Sensitivity	Specificity	F1	AUC
AMI < 1 month						
Random forest	0.915	0.916	0.915	0.882	0.915	0.915
Logistic regression	0.868	0.885	0.868	0.766	0.867	0.868
SVC	0.631	0.635	0.631	0.538	0.627	0.631
KNN	0.865	0.880	0.865	0.766	0.864	0.865
All-cause mortality < 1 month						
Random forest	0.999	0.999	0.999	1.000	0.999	0.999
Logistic regression	0.716	0.717	0.716	0.690	0.716	0.716
SVC	0.656	0.660	0.656	0.584	0.654	0.656
KNN	0.969	0.971	0.969	0.940	0.969	0.969

*SVC* support-vector clustering; *KNN* K-nearest neighbors; *ED* emergency department; *F1* 2 x (precision x recall/precision + recall); *AUC* area under the curve; *AMI* acute myocardial infarction

We identified 3741 new ED patients with chest pain between Jan 1, 2019 and Oct 31, 2019 to validate the AI prediction model (Table 4). The AUCs of AMI < 1 month and all-cause mortality < 1 month were 0.907 and 0.888, respectively. Between June 1, 2019 and May 31, 2020, the use rate of AI prediction model was 12.4% with a satisfaction of 4.98 ± 0.15 by 5-point Likert scale.

**Table 4:** Validation of the AI prediction model with new ED patients with chest pain (*n* = 3741)

Outcome	Accuracy	Precision	Sensitivity	Specificity	F1	AUC
AMI < 1 month	0.907	0.908	0.929	0.885	0.907	0.907
All-cause mortality < 1 month	0.888	0.908	0.775	0.999	0.886	0.888

*ED* emergency department; F1, 2 x (precision x recall/precision + recall); *AUC* area under the curve; *AMI* acute myocardial infarction

## Discussion

We used a big-data-driven approach, ML, and integration with HIS to build a real-time prediction for MACE in ED patients with chest pain. In the prospective validation, the AI prediction model has an AUC of 0.907 for AMI < 1 month and an AUC of 0.888 for all-cause mortality < 1 month. Compared with other ML algorithms, the random forest model had better accuracy than logistic regression, SVC, and KNN. Validation of the AI prediction model using new patients also showed excellent accuracy.

Risk stratification and disposition of ED patients with chest pain is always a difficult challenge for ED physicians, especially in overcrowding situations [6, 7, 16]. A missed diagnosis of AMI is a nightmare for ED physicians; therefore, developing a clinical prediction rule becomes a useful method to assist with decision-making and disposition [16]. The Thrombolysis In Myocardial Infarction (TIMI) score was originally developed to predict adverse cardiac outcomes (death, [re]infarction, or recurrent severe ischemia requiring revascularization) within 14 days of presentation for patients with unstable angina or non–ST-segment-elevation myocardial infarction [17]. The TIMI score was also used to predict MACE in ED patients with chest pain [18]. Another famous tool, the GRACE score, was also developed in patients already diagnosed with AMI [19]. In contrast to the TIMI and GRACE scores, the HEART score was developed more specifically for rapid risk stratification and disposition in ED patients with chest pain [4]. The HEART score has the benefits of ease of recall and use, ready availability of predictors, a focus on short-term outcomes, specificity for ED management, and identification of three risk groups [20]. Compared with the TIMI and GRACE scores, the HEART score performed better to discriminate MACE in ED patients with chest pain [21].

The real-time AI prediction model in the present study has good accuracy for risk stratification in ED patients with chest pain. In addition, its features of automatic and rapid capture of the predictors from EMRs and improving ML algorithms provide ED physicians with new hope for improving care in this population. The random forest model adopted in the present study is a common algorithm for predicting outcomes and selecting predictors in the ED [13, 22]. A study reported that ML outperformed CURB-65, MEDS, and mREMS as well as traditional analytical techniques for predicting in-hospital mortality in ED patients with sepsis [22]. Another study reported that ML-based variable selection is promising method of discovering relevant and significant predictors of MACE in ED patients with chest pain [13]. In addition to the random forest model, we used the SMOTE algorithm, an oversampling approach, to adjust imbalanced data in the present study [23–25]. In the SMOTE algorithm, the minority group is over-sampled by creating synthetic examples [23–25]. The SMOTE is suggested to be better than the under-sampling approach, which has the drawback of disregarding potentially useful data [23–25].

Implementation of AI prediction model in healthcare raises many new issues, including malpractice liability, insurance coverage, and EMR platforms. Legal issues include technology manufacturers’ and health care professionals’ liability, particularly if they can’t explain the suggestions generated by AI prediction model [26]. New legal doctrine is necessary for AI-related medical malpractice [26]. For healthcare insurance, a clear and effective data governance framework is critical [27]. In the design and use of EMR and AI, legal standards need to be enacted and insurance company should be encouraged to adopt a human-centered approach [27]. Regulators and policy-makers and insurance company need to work together to ensure that the big-data driven AI are transparent and accurate [27].

The major strength of this study is that we developed the first real-time AI prediction model integrated with the HIS for predicting MACE in ED patients with chest pain. Another strength was that we identified new patients to validate the AI prediction model. The limitations are as follows. First, we did not include electrocardiography in the analyzes due to the difficultly of interpreting unstructured data. However, our AI prediction model using structured data only performed well. Further studies by including electrocardiography may be needed to evaluate the additional value. Second, ML has the problems of interpretability and inferences about variables [28]. However, we believe that the random forests used in this study provide for good interpretability through the feature importance plots and the individual trees. Third, some data were not available due to the retrospective nature of this study. We used expert consultation to decide how to manage missing values. Fourth, we did not evaluate the impact of the AI prediction model on clinical practice. Although it is beyond the scope of this study, it may be necessary to evaluate the impact on physicians’ decisions, including acceptance and rejection, skepticism, concerns, change their clinical practices, and patient outcomes. Fifth, we used ICD-9-CM of chest pain to recruit the patients into this study, which may exclude patients who presented with chest pain but had, for instance, AMI. However, patients with immediate diagnosis of AMI are not the target population for the AI prediction model (e.g., significant ST elevation on the electrocardiography). The main difficulty is evaluating the patients with equivocal finding in the initial presentation. Therefore, the AI prediction model may play the important role to assist ED physicians for making decision in such a circumstance. Sixth, the AI prediction model may not be generalizable to other hospitals. However, this study was supposed to be a proof of concept of using AI for this purpose. We also suggest the same algorithm might be used at other institutions. Re-training and testing in other hospitals are suggested to overcome this issue.

## Conclusions

We integrated the first real-time AI prediction model with the HIS to predict short-term MACE in ED patients with chest pain. The AI prediction model has excellent accuracy and a rapid response for assisting ED physicians in decisions and disposition. Validation on new patients also showed excellent performance. Further studies about the impact of an AI prediction model on clinical practice and outcomes, as well as including electrocardiography in the analyzes, may be needed.

## Supplementary information

**Additional file 1: Supplementary Figure 1.** Feature importance according to a random forest model for predicting AMI < 1 month in ED patients with chest pain. AMI, acute myocardial infarction; ED, emergency department.

**Additional file 2: Supplementary Figure 2.** Feature importance according to a random forest model for predicting all-cause mortality < 1 month in ED patients with chest pain. AMI, acute myocardial infarction; ED, emergency department.

**Additional file 3: Supplementary Figure 3.** Screenshot of the real-time AI prediction model in the HIS for predicting MACE in ED patients with chest pain. AI, artificial intelligence; HIS, hospital information system; MACE, major adverse cardiac events; ED, emergency department.

## Data Availability

Due to the nature of this research, participants of this study did not agree for their data to be shared publicly, so supporting data is not available.

## Abbreviations

AI: Artificial intelligence
ML: Machine learning
HIS: Hospital information system
MACE: Major adverse cardiac events
ED: Emergency department
AMI: Acute myocardial infarction
SVC: Support-vector clustering
KNN: K-nearest neighbor
AUC: Areas under the curves
ACS: Acute coronary syndrome
HEART: History, Electrocardiography, Age, Risk Factors, Troponin
EMRs: Electronic medical records
ICD-9-CM: International Classification of Diseases, Ninth Revision, Clinical Modification
BMI: Body mass index
SMOTE: Synthetic minority oversampling technique
SD: Standard deviation
TIMI: Thrombolysis In Myocardial Infarction
