# Supplementary figures and images for: Real-time AI prediction for major adverse cardiac events in emergency department patients with chest pain
Source: Scand J Trauma Resusc Emerg Med. 2020 Sep 11;28:93. doi: 10.1186/s13049-020-00786-x (PMC7488862; doi:10.1186/s13049-020-00786-x)

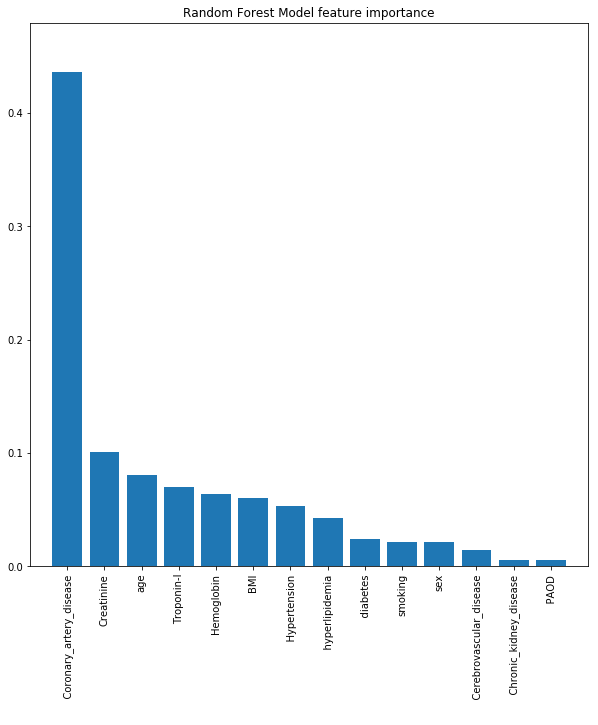

Supplement: Supplementary file 1 — Additional file 1: Supplementary Figure 1. Feature importance according to a random forest model for predicting AMI < 1 month in ED patients with chest pain. AMI, acute myocardial infarction; ED, emergency department. [file 13049_2020_786_MOESM1_ESM.tif]

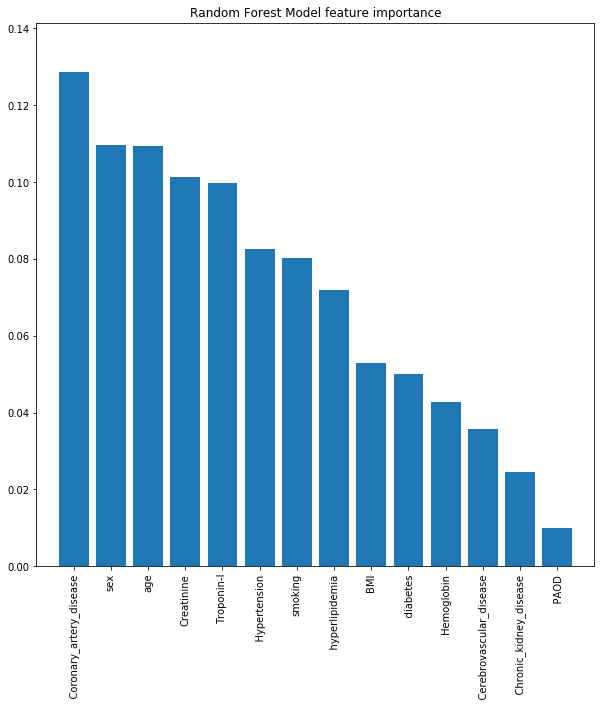

Supplement: Supplementary file 2 — Additional file 2: Supplementary Figure 2. Feature importance according to a random forest model for predicting all-cause mortality < 1 month in ED patients with chest pain. AMI, acute myocardial infarction; ED, emergency department. [file 13049_2020_786_MOESM2_ESM.tif]

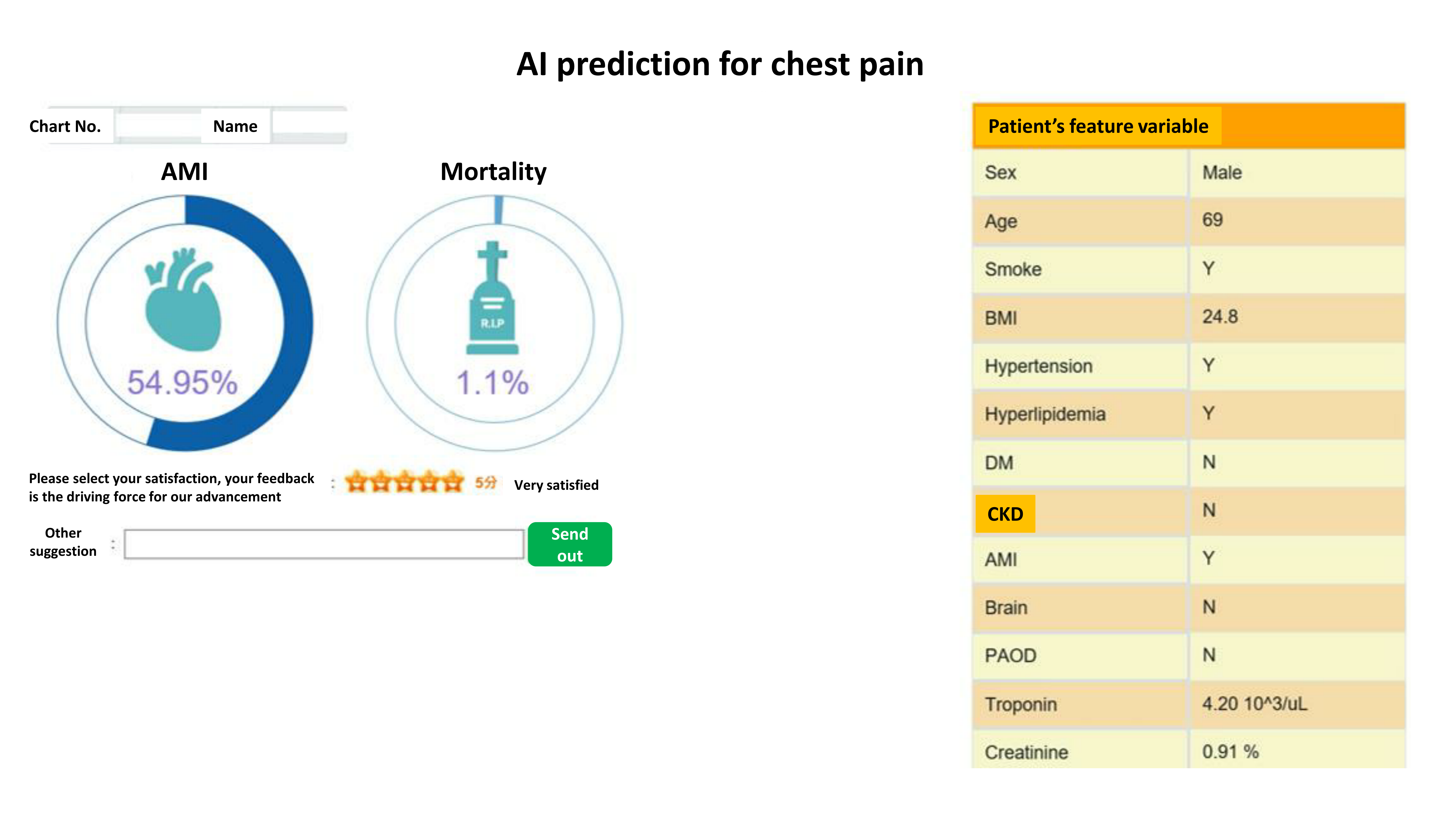

Supplement: Supplementary file 3 — Additional file 3: Supplementary Figure 3. Screenshot of the real-time AI prediction model in the HIS for predicting MACE in ED patients with chest pain. AI, artificial intelligence; HIS, hospital information system; MACE, major adverse cardiac events; ED, emergency department. [file 13049_2020_786_MOESM3_ESM.tif]
